# Supplementary figures and images for: Using trace elements to identify the geographic origin of migratory bats
Source: PeerJ. 2020 Oct 19;8:e10082. doi: 10.7717/peerj.10082 (PMC7580586; doi:10.7717/peerj.10082)

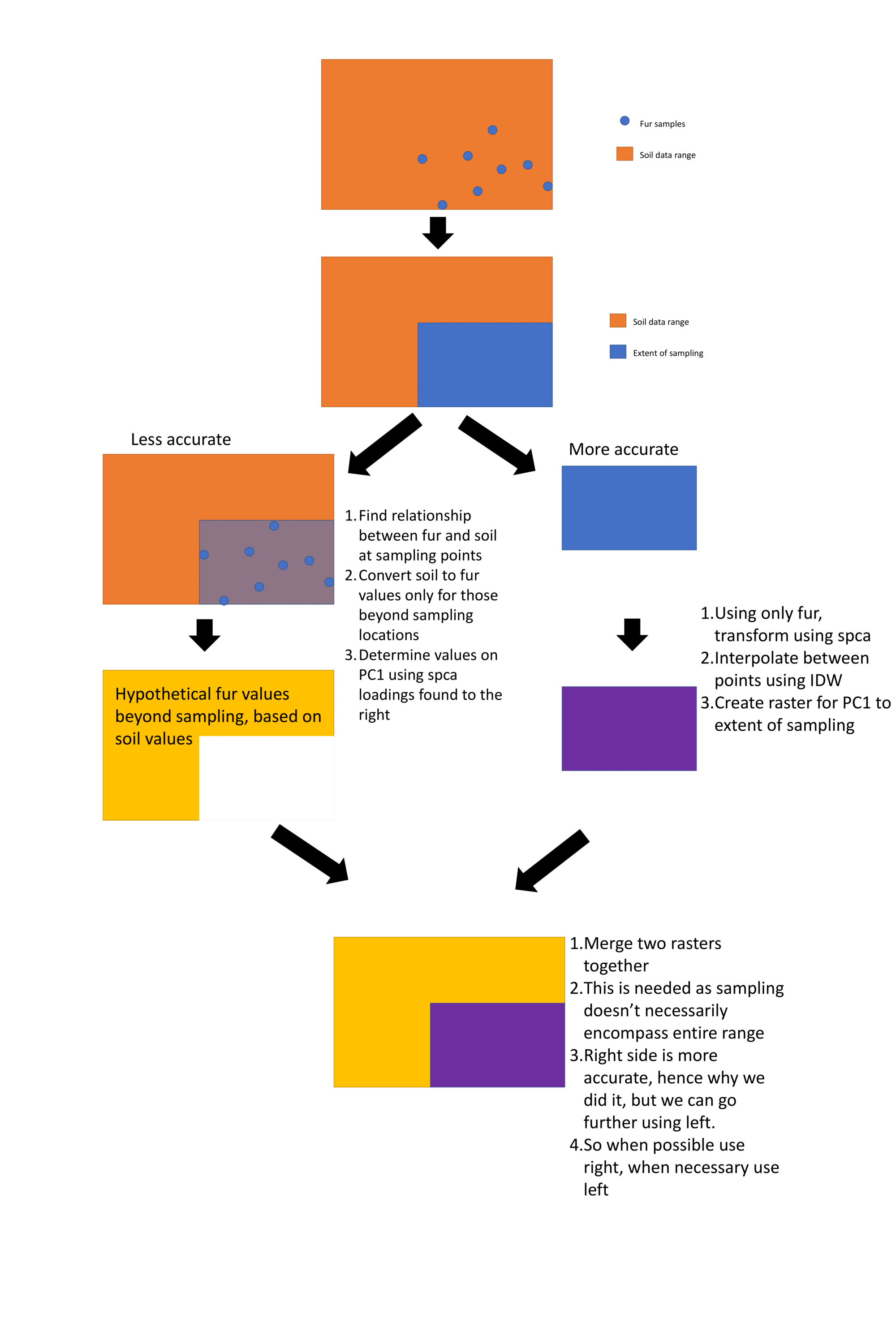

Supplement: Supplemental Information 1 [file peerj-08-10082-s001.png]

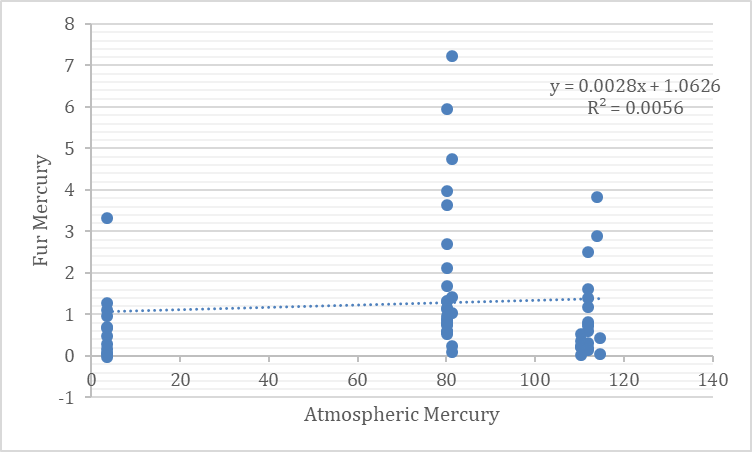

Supplement: Supplemental Information 2 [file peerj-08-10082-s002.png]

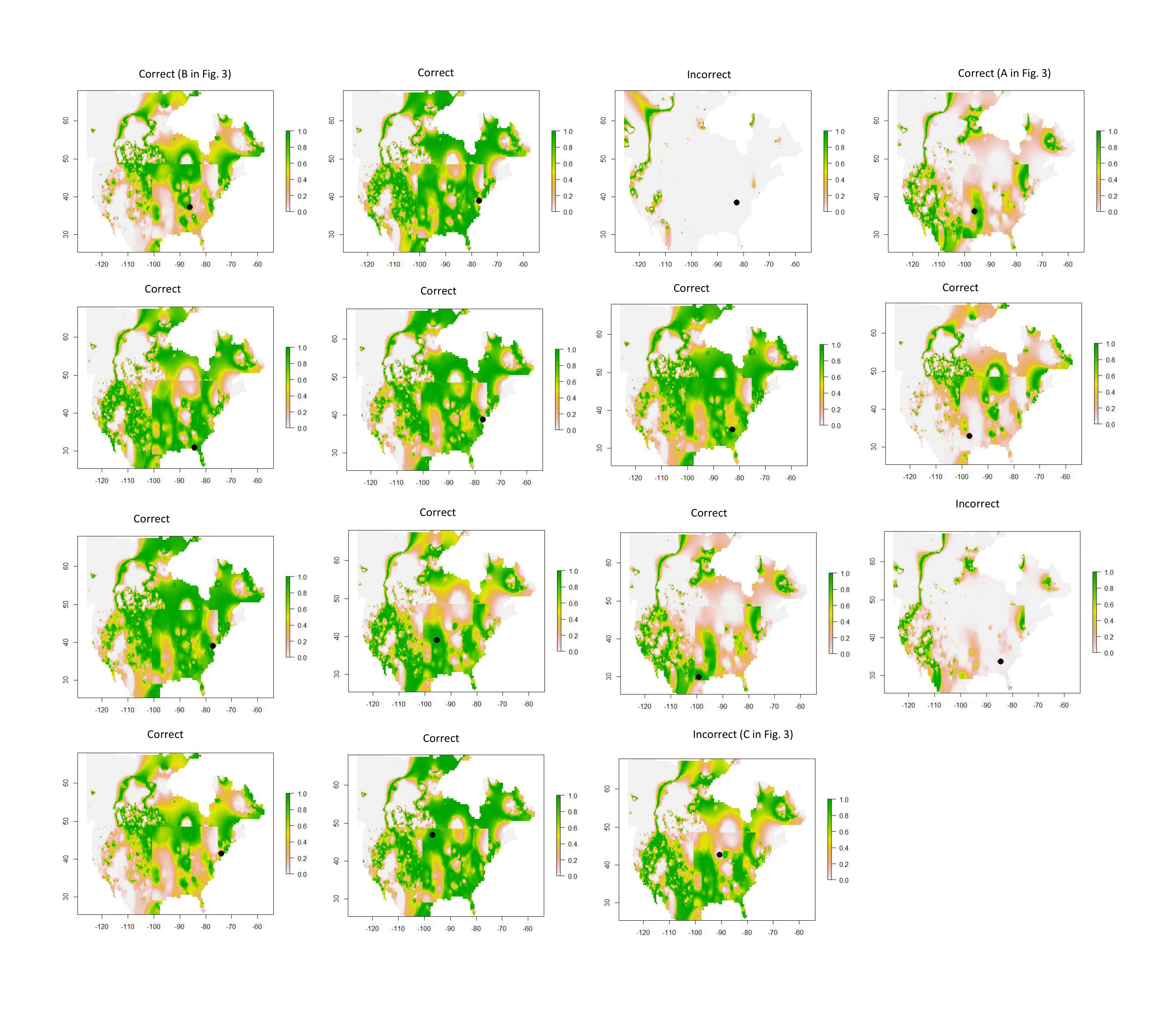

Supplement: Supplemental Information 3 — For all (n=15) independent samples, the probability surface is shown. These are in the same order as the samples listed in Table 3, and origin of the sample given by a black dot. Each sample is also listed as either ”correct” or ”incorrect”. Map outline of countries generated using ’maps’ R package (Becker et al., 2018). [file peerj-08-10082-s003.png]
